# Volatile Organic Compounds Enhance Allergic Airway Inflammation in an Experimental Mouse Model

**DOI:** 10.1371/journal.pone.0039817

**Published:** 2012-07-03

**Authors:** Ulrike Bönisch, Alexander Böhme, Tibor Kohajda, Iljana Mögel, Nicole Schütze, Martin von Bergen, Jan C. Simon, Irina Lehmann, Tobias Polte

**Affiliations:** 1 Department of Environmental Immunology, UFZ – Helmholtz Centre for Environmental Research Leipzig-Halle, Leipzig, Germany; 2 Department of Dermatology, Venerology and Allergology, Leipzig University Medical Center, University of Leipzig, Leipzig, Germany; 3 Department of Metabolomics, UFZ – Helmholtz Centre for Environmental Research Leipzig-Halle, Leipzig, Germany; University Hospital Freiburg, Germany

## Abstract

**Background:**

Epidemiological studies suggest an association between exposure to volatile organic compounds (VOCs) and adverse allergic and respiratory symptoms. However, whether VOCs exhibit a causal role as adjuvants in asthma development remains unclear.

**Methods:**

To investigate the effect of VOC exposure on the development of allergic airway inflammation Balb/c mice were exposed to VOCs emitted by new polyvinylchloride (PVC) flooring, sensitized with ovalbumin (OVA) and characterized in acute and chronic murine asthma models. Furthermore, prevalent evaporated VOCs were analyzed and mice were exposed to selected single VOCs.

**Results:**

Exposure of mice to PVC flooring increased eosinophilic lung inflammation and OVA-specific IgE serum levels compared to un-exposed control mice. The increased inflammation was associated with elevated levels of Th2-cytokines. Long-term exposure to PVC flooring exacerbated chronic airway inflammation. VOCs with the highest concentrations emitted by new PVC flooring were N-methyl-2-pyrrolidone (NMP) and 2,2,4-trimethyl-1,3-pentanediol diisobutyrate (TXIB). Exposure to NMP or TXIB also increased the allergic immune response in OVA-sensitized mice. *In vitro* or *in vivo* exposure to NMP or TXIB reduced IL-12 production in maturing dendritic cells (DCs) and enhanced airway inflammation after adoptive DC transfer into Balb/c mice. At higher concentrations both VOCs induced oxidative stress demonstrated by increased isoprostane and glutathione-S-transferase-pi1 protein levels in the lung of non-sensitized mice. Treatment of PVC flooring-exposed mice with N-acetylcysteine prevented the VOC-induced increase of airway inflammation.

**Conclusions:**

Our results demonstrate that exposure to VOCs may increase the allergic immune response by interfering with DC function and by inducing oxidative stress and has therefore to be considerate as risk factor for the development of allergic diseases.

## Introduction

Over the last two decades the prevalence of allergic diseases and asthma has increased substantially in industrial nations [1]. Although the development of atopic diseases depends on genetic background of individuals, there is growing consensus that genetic predisposition factors alone do not adequately account for such rapid shift in global prevalence [2]. Thus, altered environmental and lifestyle conditions in western civilization are thought to be responsible for the increase in prevalence of allergic diseases. Since many individuals in industrialized nations spend most of their time indoors, the quality of indoor air is of great importance [3]. In particular, it has been suggested that exposure to chemicals commonly found indoors could exhibit a role for adverse allergic airway symptoms [4]. Interior products such as synthetic coated furniture, carpets, polyvinylchloride (PVC) flooring but also building materials and cleaning agents emit an array of volatile organic compounds (VOCs) [5]. Several epidemiological studies indicate an association between exposure to indoor VOCs and adverse respiratory symptoms [6], [7] as well as an increased risk for allergic manifestations [8]–[11]. However, these studies are mainly descriptive and the specific risk factors identified may only be indicators for causal exposures. Furthermore, reviews of studies investigating allergy-promoting effects of chemical exposures in indoor environments provided only limited support for an important adjuvant effect by VOC exposure [4], [12]. Therefore, the role of indoor VOCs as irritants or adjuvants in asthma development remains an open question. However, several *in vitro* studies demonstrate that exposure to indoor relevant concentrations of VOCs increase the production of inflammatory cytokines in human lung epithelial cell lines by the induction of oxidative stress [13]–[15]. A similar pro-inflammatory cytokine pattern with increased levels of IL- 6 or TNF-alpha was found in blood samples of children after indoor renovation activities, in particular in the presence of new floor covering [16]. In mouse studies it has been shown that gaseous formaldehyde or low molecular weight respiratory sensitizers may affect allergen-specific serum IgE or IL-4 levels, respectively [17], [18]. These data suggest a direct interaction of VOCs with redox-sensitive pathways that may lead to a modified immune response. To establish a conductive relationship between exposure to indoor VOCs and the development of respiratory or allergic symptoms, we exposed mice directly to VOCs evaporated by new PVC flooring and investigated their effect on the allergic immune response in a murine asthma model. Furthermore, we analyzed and identified the emitted VOCs and measured the influence of two selected single VOCs on asthma development in mice. Our data demonstrate that PVC flooring emit a variety of VOCs and that exposure of mice to PVC flooring as well as to identified single VOCs enhanced allergic airway inflammation. The results indicate an adjuvant effect of VOC exposure that was mediated by modulating the Th1/Th2 balance via direct effects on IL-12 production in DCs and by inducing oxidative stress in the airways.

## Methods

### Mice

Female BALB/cByJ mice (6–8 weeks of age) were obtained from the Elevage Janvier Laboratory (Le Genest St Isle, France). BALB/C-Tg(NFκB-RE-luc Oslo) mice were purchased from Caliper Life Sciences (Hopkinton, USA). NFκB/luc Tg mice, which are on Balb/c background, carries a transgene containing 3 NFκB response element sites (RE) from the Igκ light chain promoter and modified firefly luciferase cDNA (Promega pGL-3). Mice were bred and maintained in the animal facility at the University of Leipzig (Germany) and housed under conventional (Balb/c) or under pathogen-free (NFκB/luc Tg) conditions with 23°C room temperature, 60% humidity, and 12 h day/night rhythm. Cages were bedded with LIGNOCEL***®*** bedding material (fine particles <200 µm 0.2%). Mice received conventional mouse feed (Altromin, Lage, Germany) or protein-rich diet (for NFκB/luc Tg, Ssniff, Soest, Germany) and water *ad libitum*. All animal experiments involved groups of 4–6 mice/cage and were performed at least 2 times according to institutional and state guidelines. The Committee on Animal Welfare of Saxony approved animal protocols used in this study.

### Immunization and Exposure to PVC Flooring

To induce acute airway inflammation Balb/c mice were immunized intraperitoneally (i.p.) with OVA (20 µg, Sigma-Aldrich, Steinheim, Germany) adsorbed to 2 mg of an aqueous solution of aluminum hydroxide and magnesium hydroxide (Alum, Perbio Science, Bonn, Germany) on days 1 and 14 followed by 20 µg OVA in 40 µl normal saline given intranasally (i.n.) on days 14 and 17–19. Control mice received Alum i.p. and normal saline i.n. AHR was measured on day 20 and mice were sacrificed on day 21. Mice were exposed to PVC flooring by installation of 20×25 cm new PVC flooring in the top cover of the mice cage to avoid direct contact to the PVC flooring. VOCs emitted by PVC flooring were measured as described in the supporting information (File S1). Mice were exposed to PVC flooring from day 0 to 20. The antioxidant N-acetylcysteine (NAC, 1 g/l) was given directly to the drinking water and adjusted to pH 7 [19]. To induce a chronic asthma-like phenotype, Balb/c mice were immunized with OVA on days 1 and 14 and challenged with OVA twice per week for 8 weeks [20]. Mice were exposed to PVC flooring over the whole period until measurement of AHR on day 71. Mice were sacrificed the following day.

### Exposure to TXIB or NMP

Animals were housed in a 300-l whole body exposure chamber (TSE Systems, Bad Homburg, Germany) with an air flow of 50 l/min. VOC exposure were performed 5 hours/day with an average mass concentration of 19±4 µg/m^3^ or 51±11 µg/m^3^ NMP and 9±2 µg/m^3^ or 32±6 µg/m^3^ TXIB. VOC concentrations were measured as described in the supporting information (File S1). Mice were exposed to NMP or TXIB either from day 0 to day 19 or only from day 17–19 to analyze the effect of VOC exposure on an ongoing allergic inflammation in sensitized mice. Control mice were exposed to ambient air.

### Measurement of Airway Responsiveness

Lung resistance (R_L_) and dynamic compliance (C_dyn_) were measured by invasive plethysmography (EMKA TECHNOLOGIES) in response to inhaled methacholine (Sigma-Aldrich) as described previously [21], [22]. Therefore, mice were anesthetized (100 mg/kg ketamine and 10 mg/kg xylazine, Bayer, Leverkusen, Germany), intubated, and mechanically ventilated at a tidal volume of 0.2 ml and a frequency of 150 breath/min. Responses to aerosolized saline (0.9% NaCl) as baseline R_L_, C_dyn_ and responses were measured first, followed by responses to increasing doses (2.5 to 40 mg/ml) of aerosolized methacholine.

### Collection of Bronchoalveolar Lavage (BAL) Fluid

Cells in the lavage fluid were counted using a hemocytometer and BAL cell differentials were determined as described previously [21], [23].

#### Lung histology and computer-based quantification of inflammation, extracellular matrix deposition and airway smooth muscle proliferation

Left lung was fixed in 10% formalin and stained with Haematoxylin & Eosin (H&E, MERCK, Darmstadt, Germany). For quantification and objective evaluation of the degree of histological inflammation, lung sections were scanned with a digital camera (Zeiss, 5 shots per lung) and analyzed with HistoClick-Software based on morphometric image analysis [20], [21]. The degree of inflammation is expressed by the number of pixels which correlate to the stained cells of interest. Lung sections were also immunostained with anti-α-smooth muscle actin (SMA) primary Ab to distinguish smooth muscle cells and with proliferating cell nuclear antigen (PCNA) to stain proliferating smooth muscle cells [20]. The primaries Abs were detected with a HRP-labeled secondary Ab (both Abcam, USA). Digital photographs of four bronchioles per tissue section were taken and analyzed with HistoClick-Software.

#### Collagen analysis

Collagen content was measured in lung tissue homogenates by a biochemical assay according to the manufacturer’s instructions (Sircol collagen assay, Biocolor, Ireland). Lung tissue (100 mg) was homogenized in 1 ml Tris buffer containing 1 M sodium chloride and a protease inhibitor cocktail (Sigma-Aldrich, Taufkirchen, Germany). Samples were incubated overnight at 4°C with stirring, centrifuged, and the supernatant was assayed [20].

### OVA-specific IgE Assay

OVA-specific IgE serum levels were measured by sandwich ELISA according to a standard protocol as described previously [21].

### Cytokine Production

Splenocytes or mediastinal lymph node cells (5×10^6^ cells/ml per well) were isolated and re-stimulated in vitro with 200 µg/ml OVA in culture medium (RPMI medium supplemented with 10% FCS, 100 U/ml Penicillin, 100 µg/ml Streptomycin) one day after airway function test. After three days cytokines were measured in supernatants from re-stimulated spleen cells or from lung tissues using DuoSet® ELISA kits (R&D Systems, Minneapolis, USA) according to the manufacturer’s instructions.

### Statistical Analysis

Statistical differences between two different groups were evaluated using Student’s *t* test. One-way ANOVA and Bonferroni’s multiple comparison tests were used to determine the statistical significance of differences between groups. Data were expressed as mean ± SEM. *P* values of less than 0.05 were considered significant.

## Results

### Emission of VOCs by PVC Flooring

We chose ordinary PVC flooring ordered directly by several companies in closed sample packages as source for indoor VOCs. A total of 24 different PVC floorings were compared in regard to their emission of VOCs. The identification and quantification of 87 different VOCs revealed several high evaporating products (Fig. S1). We identified eleven VOCs which dominated the total VOC emission. For subsequent experiments we selected one of the high evaporating PVC floorings (number 9) and analyzed the emission of VOCs. We detected eight VOCs with concentrations of more than 1 µg/m^3^ (Table 1). The VOCs with the highest concentrations were NMP (18.25 µg/m^3^) and TXIB (9.21 µg/m^3^). However, the variation of the VOC concentrations emitted by the different sample packages of the selected PVC flooring was in part extensive with maximum total VOC values of 384 µg/m^3^ and minimum values of 48 µg/m^3^ (Table 1). Furthermore, the emitted VOC concentrations diminished with the time with total VOC value of less than 45% on day 75 (single VOCs <1.4 µg/m^3^) compared to day 1 (Table 2).

**Table 1:** VOCs emitted by PVC flooring.

VOCs (µg/m^3^)	median	25^th^pctile	75^th^pctile	maximum	minimum
**NMP**	18.25	7.06	38.7	210.6	2.37
**TXIB**	9.21	2.30	64.27	117.0	1.13
**EGMP**	5.93	4.08	7.18	7.9	1.1
**DEGMB**	4.66	2.23	10.22	14.14	2.16
**2-Ethyl-1-hexanol**	3.61	3.18	5.91	8.31	1.58
**Tridecan**	2.75	0.17	5.19	6.85	0.02
**Phenol**	1.17	0.68	1.64	21.08	0.41
**Undecan**	1.08	0.75	1.49	5.51	0.59
**Σ VOC**	109.52	95.1	146.12	384.6	48.94
**79 VOC**	<1.0				

New PVC flooring was installed on the top cover of the mice cage. VOCs emitted by PVC flooring were measured on day 1 as described in the supporting information (File S1).

**Table 2:** Long-term emission of VOCs by PVC flooring.

	**Median (25^th^ and 75^th^ pctile)**					
**VOCs (µg/m^3^)**	**day 1**	**day 5**	**day 15**	**day 20**	**day 45**	**day 75**
**NMP**	18.25 (7.06–38.7)	4.87 (2.74–10.07)	4.03 (2.07–5.1)	2.94 (2.5–3.63)	1.89 (1.54–2.59)	0.66 (0.43–0.83)
**TXIB**	9.21 (2.3–64.27)	4.05 (2.49–26.1)	2.79 (2.65–20.6)	1.70 0.92–9.85)	1.35 (0.75–2.17)	1.16 (0.96–9.86)
**EGMP**	5.93 (4.08–7.18)	4.75 (3.7–9.62)	2.96 (2.88–7.08)	2.96 (2.8–4.75)	2.71 (2.58–3.13)	1.71 (0.76–2.16)
**DEGMB**	4.66 (2.33–10.22)	1.90 (1.44–2.4)	1.73 (1.38–2.04)	1.50 (1.25–1.73)	1.38 1.21–1.83)	0.74 (0.54–0.76)
**2-Ethyl-1-Hexanol**	3.61 (3.18–5.91)	3.58 (2.39–4.72)	1.85 (1.18–2.47)	1.82 (1.5–2.53)	1.70 (1.1–2.04)	0.89 (0.45–1.3)
**Tridecan**	2.75 (0.17–5.19)	2.29 (1.35–3.25)	0.31 (0.3–0.57)	0.26 (0.13–0.61)	0.23 (0.16–0.33)	0.17 (0.04–0.29)
**Phenol**	1.17 (0.68–1.64)	0.92 (0.71–1.07)	0.89 (0.81–1.24)	0.78 (0.72–1.08)	0.67 (0.52–0.91)	0.57 (0.46–0.63)
**Undecan**	1.08 (0.75–1.49)	0.94 (0.94–0.94)	0.84 (0.47–0.97)	0.61 (0.19–1.04)	0.67 (0.31–0.97)	0.39 (0.32–0.41)
**Σ VOC**	109.52 (95.1–146.1)	73.00 (50.9–94.1)	70.22 (62.9–93.4)	69.35 (61.9–75.4)	62.22 (59.4–65.1)	49.08 (45.1–54.1)

New PVC flooring was installed on the top cover of the mice cage. VOCs emitted by PVC flooring were measured starting with day 1 on different time point until day 75 as described in the supporting information (File S1).

### Exposure to PVC Flooring Increases the Acute Allergic Immune Response in OVA-immunized Mice

**Figure 1:**
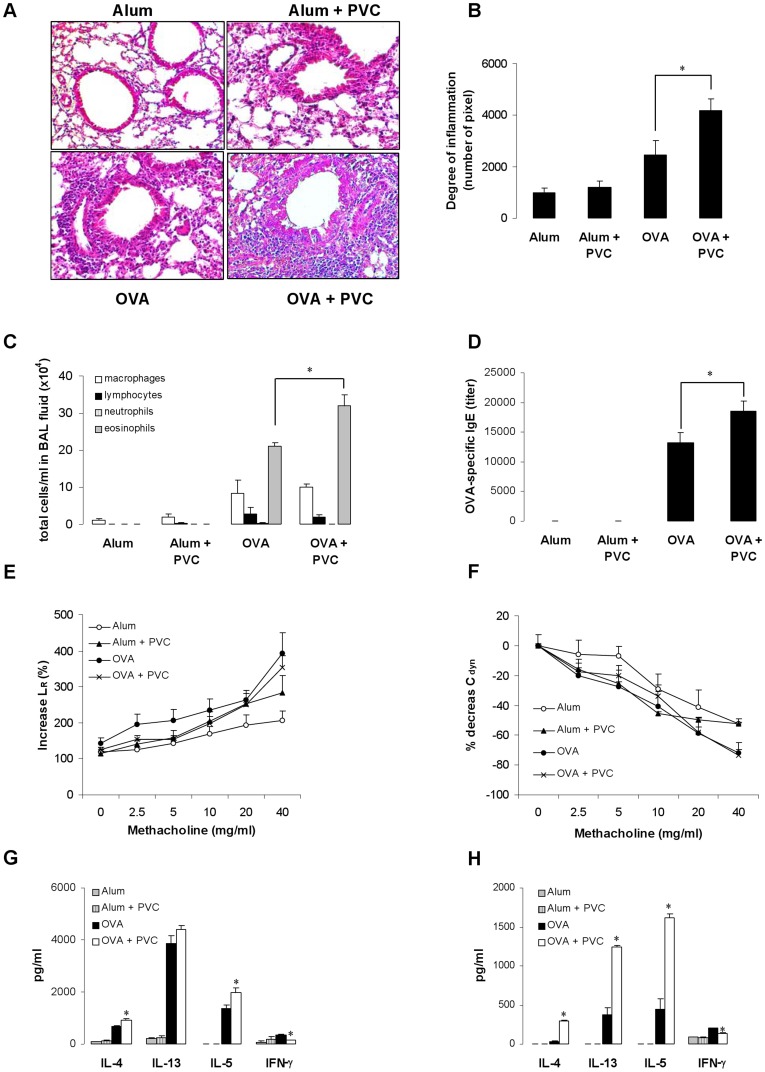
Exposure to PVC flooring increases acute airway inflammation, antigen-specific IgE and Th2 cytokine levels and reduced IFN-γ production in OVA-sensitized Balb/c mice. Balb/c mice were immunized with OVA (day 1 and 14) and then challenged with OVA on days 14 and 17–19. Mice were exposed to PVC flooring from day 0 to day 20. Effect of PVC flooring on inflammation (H&E, x200, A), verified by an objective, investigator-independent computer-based quantification of lung inflammation (B), on total cell numbers in the BAL fluid (C), OVA-specific IgE levels (D), lung resistance (E), lung compliance (F) and Th2 cytokine levels in the supernatant of OVA-re-stimulated splenocytes (G) or mediastinal lymphnodes (H). Data are expressed as mean ± SEM, n ≥ 9 animals per group; *p<0.05 *vs*. OVA.

To investigate the role of VOC exposure in the development of the allergic immune response, we exposed mice to PVC flooring in our established acute asthma model [21]–[23]. Mice exposed to PVC-VOCs with a total concentration of more than 90 µg/m^3^ revealed exacerbated airway inflammation in the lung demonstrated by an increase in inflammatory infiltrates in H&E stained lung sections (Fig. 1A), verified by objective, investigator-independent computer analysis (Fig. 1B). Exposure to PVC flooring also enhanced the number of eosinophils in the BAL fluid compared to mice that received only the antigen (Fig. 1C). In parallel, in OVA-immunized mice exposed to PVC flooring serum levels of OVA-specific IgE (Fig. 1D) were increased. Although exposure of mice to PVC flooring enhanced airway inflammation and IgE levels, significant effects on AHR were not found (Fig. 1E, F). In addition, exposure to PVC flooring increased Th2 cytokine levels, specifically IL-4 and IL-5, and decreased the production of the Th1 cytokine IFN-γ in cell culture supernatants of OVA-re-stimulated splenocytes (Fig. 1G) and mediastinal lymph node cells (Fig. 1H).

Notably, an exposure to PVC flooring with a total VOC concentration of less than 50 µg/m^3^ had no effect on the asthma phenotype.

### Long-term Exposure to PVC Flooring Exacerbates Chronic Airway Inflammation

**Figure 2:**
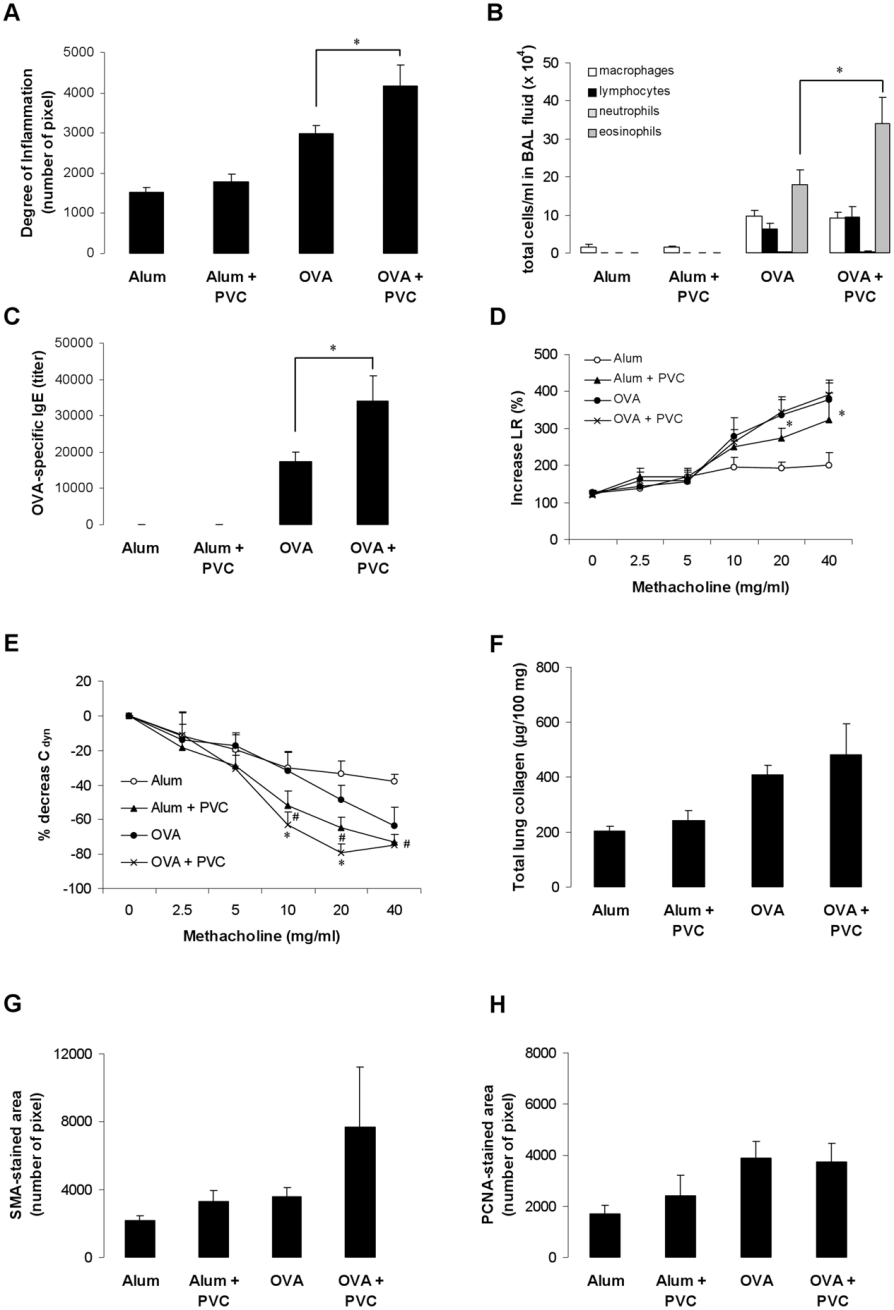
Long-term exposure to PVC flooring exacerbates chronic airway inflammation. To induce a chronic asthma phenotype mice were immunized with OVA (day 1 and 14) and then challenged with the antigen twice per week for 8 weeks. Effect of exposure to PVC flooring on inflammation of lung tissues (A), total cell numbers in BAL fluid (B), IgE levels (C), lung resistance (D), dynamic compliance (E), total lung collagen (F), and α-SMA- and PCNA-stained lung sections (G, H). Data are expressed as mean ± SEM, n ≥ 9 animals per group; *P<0.05 OVA + PVC *vs*. OVA; ^#^P<0.05 Alum + PVC *vs*. Alum.

Long-term VOC exposure significantly enhanced chronic airway inflammation and the number of eosinophils in the BAL fluid as well as OVA-specific IgE serum levels compared to control mice (Fig. 2A–C). Interestingly, in contrast to the acute model, long-term exposure to PVC flooring in the chronic model significantly increased lung resistance and decreased dynamic compliance in non-sensitized mice and dynamic compliance in OVA-sensitized mice compared to the control. (Fig. 2D, E). However**,** exposure to PVC flooring did not alter the development of airway remodelling. The amount of total lung collagen was slightly but not significantly enhanced (Fig. 2F). Furthermore, there was no significant increase on other markers for airway remodelling like the smooth muscle area (α-SMA-stained cells) or the number of proliferating cell nuclear antigen^+^-stained cells (Fig. 2G–H). Beside the effect on lung function long-term PVC exposure of non-sensitized mice showed no effect on airway inflammation, IgE levels or the development of airway remodelling (data not shown).

### NMP and TXIB Dose-dependently Inhibit IL-12 Production in LPS-stimulated Murine DCs

**Figure 3:**
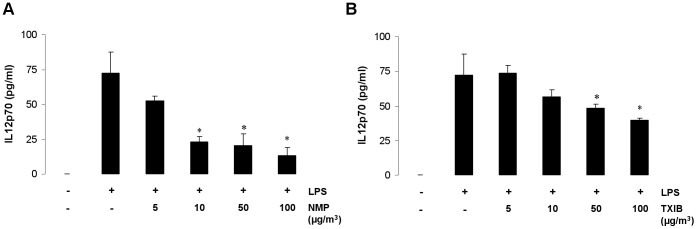
NMP and TXIB inhibit IL-12 production in LPS-stimulated murine DCs *in vitro*. Effect of exposure of LPS-stimulated BMDCs to NMP (A) and TXIB (B) on IL-12 production. Data are expressed as mean ± SEM; n = 4–5/group. *P<0.05 *vs*. LPS.

The enhanced airway inflammation after exposure to PVC flooring was accompanied by a reduced release of the Th1 cytokine IFN-γ and an increase of Th2 cytokine production (Fig. 1G, H). To investigate the effect of VOCs on DC function NMP and TXIB - the two VOCs emitted by PVC flooring at the highest concentrations - were exposed to LPS-stimulated immature murine bone-marrow-derived DCs as described in the supporting information (File S1). LPS is known to induce maturation associated IL-12 production in DCs that is important for the induction of a Th1 immune response [24]. NMP and TXIB both inhibited the LPS-induced IL-12 production in DCs in a dose-dependent manner (Fig. 3A, B). The used concentrations had no effect on cell viability as measured by trypan blue assay (data not shown).

### Exposure to NMP and TXIB Increases the Allergic Immune Response in OVA-immunized mice

**Figure 4:**
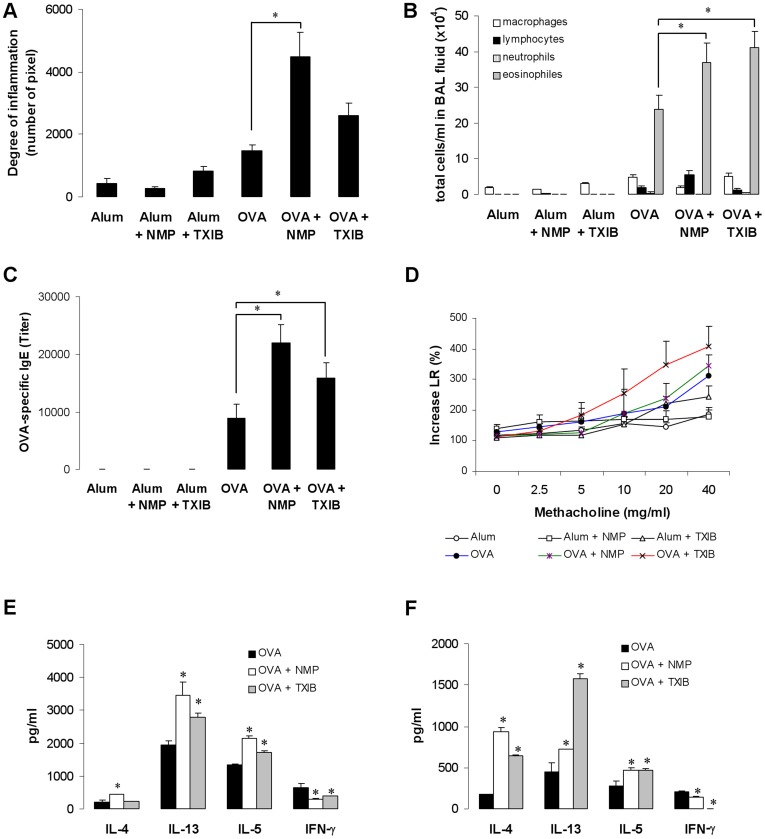
Exposure to NMP and TXIB enhances the asthma-like phenotype in OVA-sensitized Balb/c mice. Balb/c mice were immunized with OVA (day 1 and 14) and then challenged with OVA on days 14 and 17–19. Mice were exposed to NMP or TXIB from day 0 to day 20. Effect of NMP and TXIB on lung inflammation (A), total cell numbers in BAL fluid (B), IgE levels (C), lung resistance (D), and Th2 cytokine levels in the supernatant of OVA-re-stimulated splenocytes (E) or mediastinal lymphnodes (F) compared to the unexposed group. Data are expressed as mean ± SEM, n ≥ 9 animals per group; *p<0.05 *vs*. OVA.

To investigate whether exposure to single VOCs affects the allergic immune response, we exposed mice to 19 µg/m^3^ NMP or to 9 µg/m^3^ TXIB during OVA-sensitization in a whole body inhalation chamber. Both single VOCs enhanced airway inflammation (Fig. 4A), the number of eosinophils in the BAL fluid (Fig. 4B) and antigen-specific IgE levels (Fig. 4C). Whereas the lung resistance was slightly but not significantly increased (Fig. 4D), we observed elevated levels of Th2 cytokines and a reduced IFN-γ production in NMP- or TXIB-exposed OVA-sensitized mice (Fig. 4E–F). To investigate whether NMP or TXIB exposure has an effect on an ongoing allergic inflammation in already sensitized mice, we exposed mice to these VOCs only during antigen challenge. Here, exposure to 19 µg/m^3^ NMP or 9 µg/m^3^ TXIB – the concentrations showing adjuvant effects after exposure during sensitization - had no significant impact on airway inflammation, IgE- or cytokine levels (Fig. S2A–D). However, using higher but still indoor-relevant VOC concentrations (NMP: 51 µg/m^3,^ TXIB: 32 µg/m^3^) we observed elevated OVA-specific IgE serum levels as well as Th2 cytokine levels in the supernatant of OVA-restimulated lymph node cells accompanied by enhanced eosinophilic inflammation in the BAL and – at least in part – significantly increased lung resistance compared to non-exposed OVA-immunized control mice (Fig. S2A–D).

### NMP- and TXIB-induced Increase of Th2 Immune Response is Mediated by Reduced IL-12 Production of DCs

**Figure 5:**
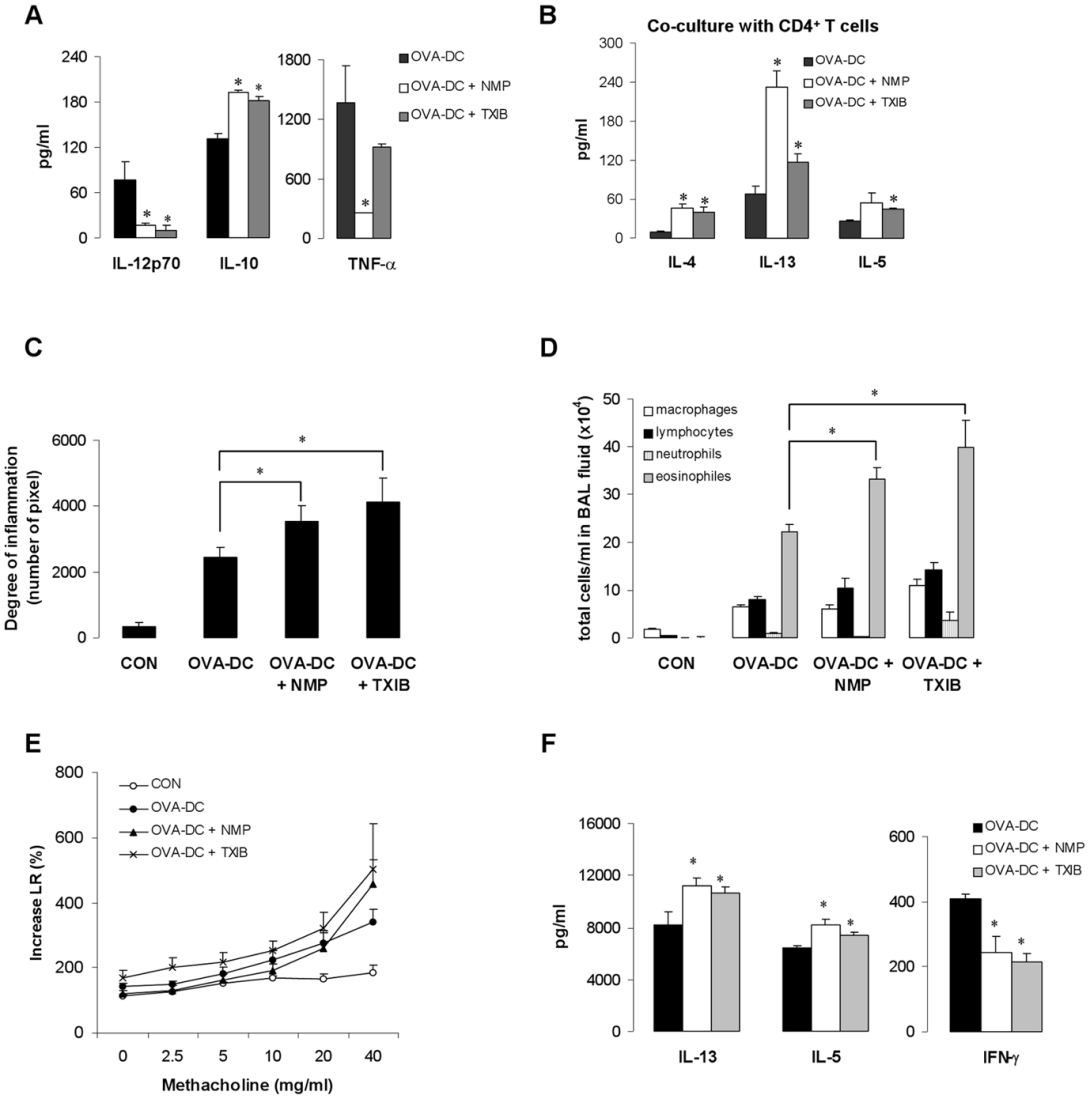
NMP- or TXIB-induced increase of Th2 immune response is mediated by reduced IL-12 production by DCs. BMDCs were pulsed for 24 h with OVA (200 µg/ml), some cells in the presence of NMP or TXIB (100 µg/m^3^), co-cultured with CD4^+^ T cells from DO11.10 mice or transferred into the trachea of naïve Balb/c mice. As control (CON) DCs only treated with LPS (100 ng/ml) was transferred. From day 10 onward, mice were challenged i.n. with OVA for 3 consecutive days. Cytokine production of DCs before transfer (A), cytokine production of DCs co-cultured with CD4^+^ T cells from DO11.10 mice (B) and effects of DC transfer on airway inflammation in H&E stained lung sections (C), total cell numbers in BAL fluid (D), lung resistance (E), and Th2 cytokine levels in the supernatant of OVA-re-stimulated mediastinal lymphnodes (F) are shown. Data are expressed as mean ± SEM, n ≥ 9 animals per group; *p<0.05 *vs*. OVA.

To verify the role of DCs and their modulated function after VOC-exposure, we pulsed DCs with OVA (Sigma, endotoxin activity: 36.04±2.80 EU/20 µg OVA which correspond to 3.6 ng LPS) and exposed the cells with NMP and TXIB (100 µg/m^3^) for 24 hours (File S1). In the supernatant of OVA (Sigma) treated DCs increased levels of IL-12 were found which were decreased after additional treatment with NMP or TXIB (Fig. 5A). Furthermore, the levels of tumor necrosis factor-alpha (TNF-α) were also decreased, whereas NMP and TXIP exposure led to an increased IL-10 production (Fig 5A). Co-cultures of OVA-pulsed DCs with DO11.10 T cells which are transgenic for T cell receptor recognizing OVA specific peptide [25] revealed an enhancement of IL, 4, IL-13 and IL-5 release when OVA-pulsed DCs were additionally exposed to NMP or TXIB (Fig. 5B). OVA-pulsed-DCs were then transferred intratracheally into naïve Balb/c mice. Mice that received NMP- or TXIB-treated DCs showed an enhanced airway inflammation (Fig. 5C), increased eosinophilic inflammation in the BAL fluid (Fig. 5D), and increased levels of Th2 cytokines IL-5 and IL- 13 in mediastinal lymphnodes whereas IFN-γ production was reduced compared to mice that received non-exposed OVA-pulsed DCs (Fig. 5F). AHR measured as lung resistance was not significantly increased (Fig. 5E).

### Exposure to NMP and TXIB May Induce Oxidative Stress in Vitro and in Vivo

**Figure 6:**
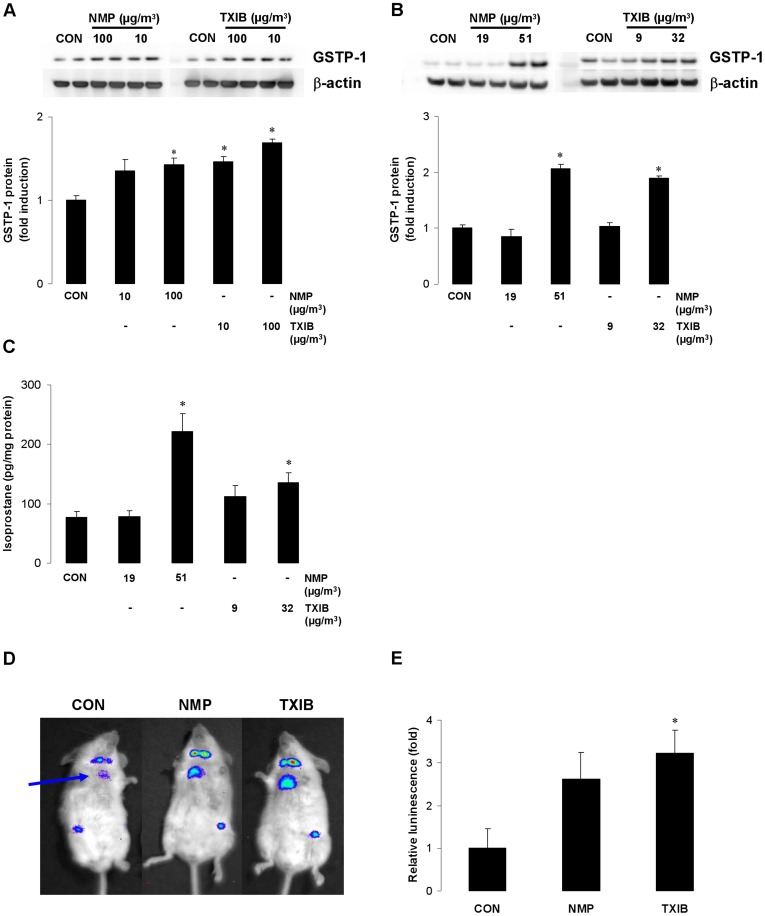
NMP and TXIB induce oxidative stress *in vitro* and *in vivo.* A549 human lung epithelial cells were exposed to NMP or TXIB for 24 h. Expression of GSTP-1 was quantified by western blotting (A). Naïve mice were exposed to NMP or TXIB on two consecutive days for 6 hours, lung tissues were taken and GSTP-1 expression was quantified by western blotting (B) and 8-isoprostane (C) was measured as described in File S1. In the pseudoimages, the blue to red coloring represents the lowest to the highest light intensity in NMP (52 µg/m^3^)- or TXIB (31 µg/m^3^)-exposed NFκB/luciferase transgenic mice (D). Photon emission was quantified over the lung area (arrow) as mean fold of induction (E). Data are expressed as mean ± SEM from three independent experiments (A), n ≥ 9 animals per group (B, C) or *n* = 4 per group (D, E); *p<0.05 *vs*. CON.

To find out whether NMP or TXIB themselves may induce oxidative stress in the lung as shown for other VOCs [15], [26] we exposed human bronchial epithelial cells (A549) to NMP and TXIB using an air-liquid cell culture system and measured the expression of glutathione-S-transferase-pi1 (GSTP-1), a cellular marker for oxidative stress (File S1). Both VOCs increased GSTP-1 protein levels compared to untreated control cells (Fig. 6A). A similar expression pattern was observed in lung tissues after short-term exposure of naïve mice to NMP or TXIB. At concentrations of 51 µg/m^3^ NMP and 32 µg/m^3^ TXIB the GSTP-1 protein levels were significantly enhanced, whereas lower VOC-concentrations had no impact (Fig. 6B). To further evaluate the role of oxidative stress, 8-isoprostane levels as marker for lipid peroxidation were analyzed in lung homogenates [27]. Short-term exposure of naïve Balb/c mice to the VOCs led to elevated isoprostane levels at the higher concentrations (Fig. 6C) but had no significant effect after exposure to lower concentrations (19 µg/m^3^ NMP, 9 µg/m^3^ TXIB). At the higher concentrations both VOCs induced lung NF-κB activation documented in NF-κB/luciferase transgenic mice *in vivo* (Fig. 6D). Additionally, NF-κB activation is also increased in submandibular lymphnodes (the reactivity in the thigh is probably induced by intramuscular injection). After 6 hours exposure NMP and TXIB increased NF-κB activation in the lung as quantified by photon emission (relative intensity units per lung area, Fig. 6E).

### NAC Reduces the Enhanced Allergic Immune Response Induced by Exposure to PVC Flooring

**Figure 7:**
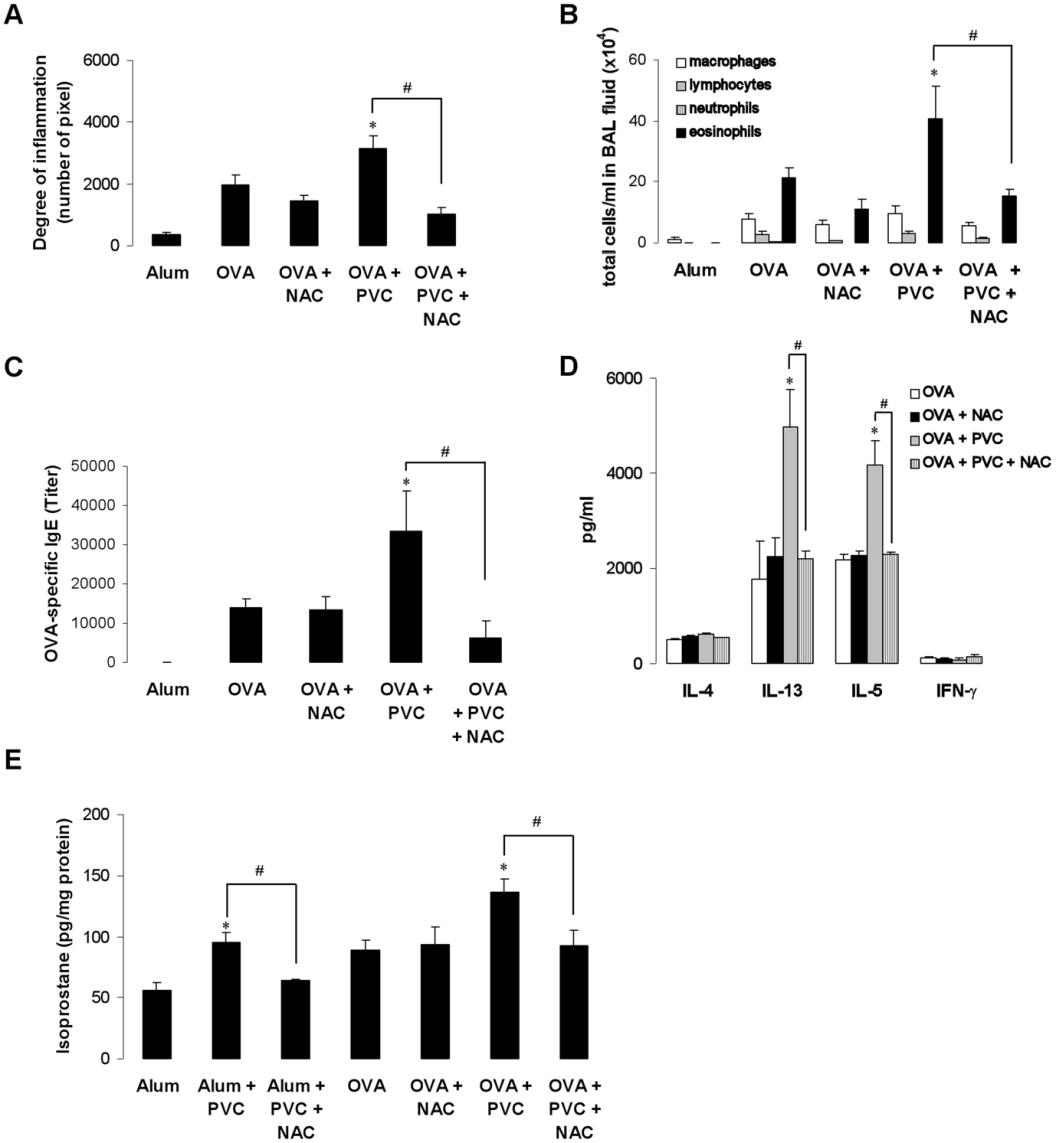
Increased acute allergic immune response by PVC flooring is abrogated by the antioxidant NAC. Treatment of Balb/c mice with NAC (1g/l) during exposure to PVC flooring reduced the enhanced acute airway inflammation (A), the number of eosinophils in the BAL fluid (B), OVA-specific IgE levels (C), IL-13 and IL-5 cytokine levels (D) and the production of 8-isoprostane in lung homogenates (E) compared to NAC-untreated mice. Data are expressed as mean ± SEM, n ≥ 9 animals per group; *P<0.05, PVC flooring *vs.* OVA; ^#^P<0.05, PVC flooring *vs*. PVC flooring + NAC.

Since the induction of oxidative stress by VOCs seems to be - at least in part - involved in the asthma-increasing effects, we added the antioxidant NAC directly into the drinking water of PVC-exposed and OVA-immunized Balb/c mice. The exacerbated allergic immune response in PVC-exposed mice compared to control mice was diminished by treatment with NAC. Similarly, airway inflammation, the number of eosinophils in the BAL fluid, OVA-specific IgE levels and cytokine levels were significantly reduced in NAC-treated mice exposed to PVC (Fig. 7A–D). In comparison to control mice allergen challenge in sensitized mice increased the isoprostane levels, which were further enhanced by exposure to PVC and abrogated in the presence of NAC (Fig. 7E). In addition, PVC exposure to non-sensitized mice led to elevated isoprostane concentrations compared to un-exposed mice. However, treatment of OVA-sensitized mice with NAC had no significant effect on asthma parameters or isoprostane levels.

## Discussion

In the present study we demonstrate in a murine asthma model that exposure to indoor VOCs emitted by PVC flooring enhanced the acute allergic immune response as well as the development of chronic airway inflammation.

Only a few epidemiological studies provide indirect evidence of the potentially adverse health effects of indoor VOCs demonstrating a positive association between renovation or painting and asthma [6], [7], [28]–[30]. These studies used indoor renovation activities as surrogates of exposure to VOCs. Although this scenario considers an exposure to increased VOC levels about at least a couple of weeks the major disadvantage is that the specific agent who might be responsible for the observed adverse health effects is not known. Epidemiological studies on measured VOCs and asthma are rare. School-based studies from Sweden reported a significant association between asthma prevalence and VOCs [31] or proposed that specific VOCs, namely propylene glycol and glycol esters, exacerbate allergic symptoms, asthma and eczema [8]. In a population-based case control study an increased risk of having asthma was shown for children exposed to VOCs [32]. However, although indirect evidence from renovation activities and direct evidence by measured VOCs point to a positive association with asthma, the level of evidence for positive association between these exposures and development of asthma is insufficient [33]. Furthermore, a causal relationship between exposure to specific VOCs and adverse health effects is still missing. Therefore, using a murine asthma model, we investigated the effect of indoor-relevant VOC on the allergic immune response by exposure mice directly to VOCs emitted by PVC flooring. PVC flooring has been shown to be associated with wheezing and allergic symptoms [29], [34]. The analysis of different PVC floorings resulted in eleven VOCs which dominated the total VOC emission. The PVC flooring used in our study was one of the high evaporating products. However, although we utilized the same type of PVC flooring for all of our experiments, the variation of the VOC concentrations emitted by the different sample packages was in part extensive. We also demonstrated that VOC concentrations diminished with time. Thus, indoor levels of VOCs emitted by PVC flooring clearly depend on kind and duration of the storage of PVC flooring after production. There is evidence to support this claim for other VOC sources such as new furniture, carpets, or building materials as well [5]. These results using PVC flooring, as an example, provide an indication for the difficulties to associate long-term VOC exposure and adverse health effects in epidemiological studies. Indoor renovation activities as surrogates of VOC exposure as well as performing just a single VOC measurement reflects the exposure conditions only partially leading to insufficient evidence [4], [33]. Notably, whereas exposure with a total VOC concentration of more than 90 µg/m^3^ increased the allergic immune response in mice compared with unexposed control animals, exposure to PVC flooring with a total VOC concentration of less than 50 µg/m^3^ had no effect on the asthma phenotype. This is comparable with a population-based case control study demonstrating a fourfold increased asthma risk for children exposed to concentrations of total VOCs of >60 µg/m^3^ [32]. Therefore, to reduce the risk for adverse health effects PVC flooring requires ventilation for some time. However, long-term exposure of sensitized mice exacerbated chronic airway inflammation even if the VOC concentration at the end of the protocol was <50 µg/m^3^. In contrast to the acute model, long-term exposure to PVC flooring significantly enhanced lung resistance and decreased dynamic compliance in non-sensitized mice and dynamic compliance in OVA-sensitized mice compared to the control. Why VOC exposure induced only moderate effects on AHR in the acute model is unclear and has to be elucidated, yet. However, similar observations with increased acute airway inflammation without affecting AHR were shown before for diesel particulate matter- or stress-induced allergic immune responses [35], [36]. Moreover, in our 12-weeks protocol we could not detect any effect of PVC flooring on airway remodeling. This could be explained by the inability of lower VOC concentrations to induce oxidative stress, known to be involved in the development of airway remodeling [37].

To understand the impact of VOC exposure under defined conditions and to characterize the underlying mechanisms we selected the two VOCs with the highest concentrations emitted by PVC flooring: NMP and TXIB. The emission of these two VOCs by PVC flooring has been described earlier [11], [38]. NMP is used for many different purposes, e.g. as solvent for a wide range of chemicals and polymer products, for stripping paint or for cleaning in the industry [39], [40]. There are some reports about occupational exposure concentrations of NMP; e.g. up to 10 mg/m^3^ in the personal breathing zones of graffiti removers [41] or up to 6 mg/m^3^ for workers in the microelectronics fabrication industry [42]. However, no information was found on NMP levels in ambient air. TXIB has been also detected in living rooms after painting [11], beside the common emission by PVC products [43]. A previous study indicated an increased risk for symptoms like nose and eye irritation after exposure to TXIB emitted by PVC flooring [44]. In a population-based study indoor TXIB concentrations were found to range from 11–71 µg/m^3^ with a maximum concentration of 373 µg/m^3^, measured in an apartment with all rooms painted and with a 1-year-old PVC floor covering [11]. Exposure to NMP or TXIB at the respective median concentration of our PVC-flooring experiments increased the allergic immune response when applied during antigen sensitization but not in already sensitized mice. Higher, but still indoor-relevant NMP or TXIB levels during the antigen challenge enhanced the allergic immune response compared to unexposed OVA-immunized control animals. These findings suggest different mechanisms to be involved. A previous population-based cohort study has shown that indoor VOC exposure induced a T cell polarization toward the type 2 phenotype [10]. It has also been demonstrated that environmental pollutants like diesel exhaust particles [45] or mycotoxins [22] may interfere with DC function leading to decreased Th1 differentiation. Therefore, we investigated the capability of both NMP and TXIB to alter IL-12 production by DCs which drives IFN-γ production in antigen-specific Th1 cells [24]. Treatment of DCs with NMP or TXIB reduced the LPS-induced IL-12 production. Moreover, NMP and TXIB decreased TNF-α levels and increased IL-10 production in the supernatant of OVA-pulsed DCs. It has been reported that a lack of DC-produced TNF-α switches inflammation from a neutrophilic to an eosinophilic bias [46], whereas DCs that produce IL-10 instead of IL-12 promote Th2 cell differentiation [47]. NMP or TXIB-treated DCs also induced an enhanced Th2 cytokine release when co-cultured with CD4 T cells from DO11.10 mice demonstrating their T cell priming capacity. To show the DC-polarizing effect of VOC exposure on the Th2–driven allergic immune response *in vivo* we transferred OVA-pulsed or additionally VOC-exposed DCs into naïve Balb/c mice. As described before [22] we used OVA containing traceable amounts of LPS since the experimental allergen OVA, which does not have any intrinsic activating properties, requires additional signals to trigger DC activation. The transfer of VOC-treated and OVA-pulsed DCs enhanced the allergic airway inflammation including Il-5 and IL-13 and reduced IFN-γ production in the lymphnodes compared to control mice. These data implicate that the decreased IFN-γ production after VOC exposure *in vivo* is specifically due to the effects of NMP and TXIB on DC populations.

Several *in vitro* studies using human alveolar epithelial cells have shown that exposure to single aromatic VOCs induces an inflammatory response via oxidative stress and NFκB activation [15], [26], [48], [49]. Oxidative stress, the imbalance between reactive oxygen species (ROS) and antioxidants, has been shown to play an important role in the pathogenesis of asthma and airway inflammation [50]. Exposure of human alveolar epithelial cells to indoor-relevant concentrations of NMP or TXIB increased protein expression of the cellular marker for oxidative stress GSTP-1 that was comparable to the mentioned studies. Short-term exposure to NMP or TXIB induced GSTP-1 protein expression in the lung at the higher concentrations just like isoprostane levels as marker for lipid peroxidation. In addition, comparable to earlier studies [26], [51] both VOCs induced the activation of NFκB as shown after exposure of NF-κB/luciferase transgenic mice. It has been shown before that immunoreactive luciferase protein is detectable in bronchiolar and alveolar epithelium as well as endothelial cells, which indicated NF-κB activation in these cells [52]. In contrast, we could not detect an influence on the expression of the pro-allergic cytokine thymic stromal lymphopoitin by VOC exposure in lung tissue (data not shown). These results implicate the induction of oxidative stress in the lung as an additional mechanism for the observed adjuvant VOC effects comparable with earlier findings investigating the role of elemental carbon ultrafine particle or mycotoxin exposure on airway inflammation [22], [27]. Whereas low concentrations of NMP and TXIB show an impact on the Th1/Th2 balance with reduced IFN-γ cytokine production after exposure during sensitization the induction of oxidative stress in the lung was only detectable at higher concentrations by exposure during the antigen challenge. However, we cannot exclude a role of ROS induced by low VOC levels leading to a modified DC function. In particular, ROS at low concentrations in its function as chemical messenger in signalling pathways could be involved without having an effect on oxidative stress markers used in our study [50], [53], [54]. At least, treatment of mice with the antioxidant NAC reduced the exacerbated allergic immune response in OVA-sensitized mice exposed to PVC flooring further supporting a role of oxidative stress. The successful use of NAC to reverse pollutant-induced immunological effects has been reported before suggesting antioxidants as alternative for health prevention [27], [55].

In conclusion, our data demonstrate a causal relationship between an exposure to indoor-relevant VOC concentrations emitted by PVC flooring and an increased allergic immune response, whereby this effect needs a threshold on total VOC levels. Therefore, the results suggest that the health risk is even more significant for occupational workers involved in the manufacture, packaging, and installation of PVC flooring. Exposure to selected single VOCs also enhanced the allerigic immune response. The observed adjuvant effect of NMP and TXIB after exposure during sensitization is mediated by interfering with maturation-associated IL-12 production in DCs and IFN-γ production in T cells leading to a decreased Th1 differentiation. Additionally, exposure to VOCs caused oxidative stress in the lung that may also enhance the allergic immune response. Taken together, our results suggest that indoor VOCs may be a risk factor for the development of allergic diseases like allergic asthma.

## Supporting Information

Figure S1 **VOC emission by different PVC floorings.** The measurement of VOCs by headspace sampling over 24 different PVC floorings and analysis with GC-MS revealed that the VOC emission is dominated by 11 VOCs out of 87 measured VOCs. For further *in vivo* experiments PVC flooring number 9 was selected.
(TIF)

Figure S2 **Effect of an exposure to NMP or TXIB during antigen challenge on asthma-like phenotype.** To analyze the effect of NMP or TXIB exposure on an ongoing allergic inflammation Balb/c mouse were sensitized and exposed to different concentrations of the VOCs as described under Methods. Effect of exposure to NMP and TXIB on total cell numbers in BAL fluid (A), OVA-specific IgE levels (B), lung resistance (C), and cytokine production in the supernatant of OVA-re-stimulated mediastinal lymphnodes (D). Data are expressed as mean ± SEM, n ≥ 9 animals per group; *P<0.05 *vs.* OVA.
(TIF)

File S1 **Supporting Information.**
(DOC)
