# Supplementary figures and images for: Volatile Organic Compounds Enhance Allergic Airway Inflammation in an Experimental Mouse Model
Source: PLoS One. 2012 Jul 3;7(7):e39817. doi: 10.1371/journal.pone.0039817 (PMC3389035; doi:10.1371/journal.pone.0039817)

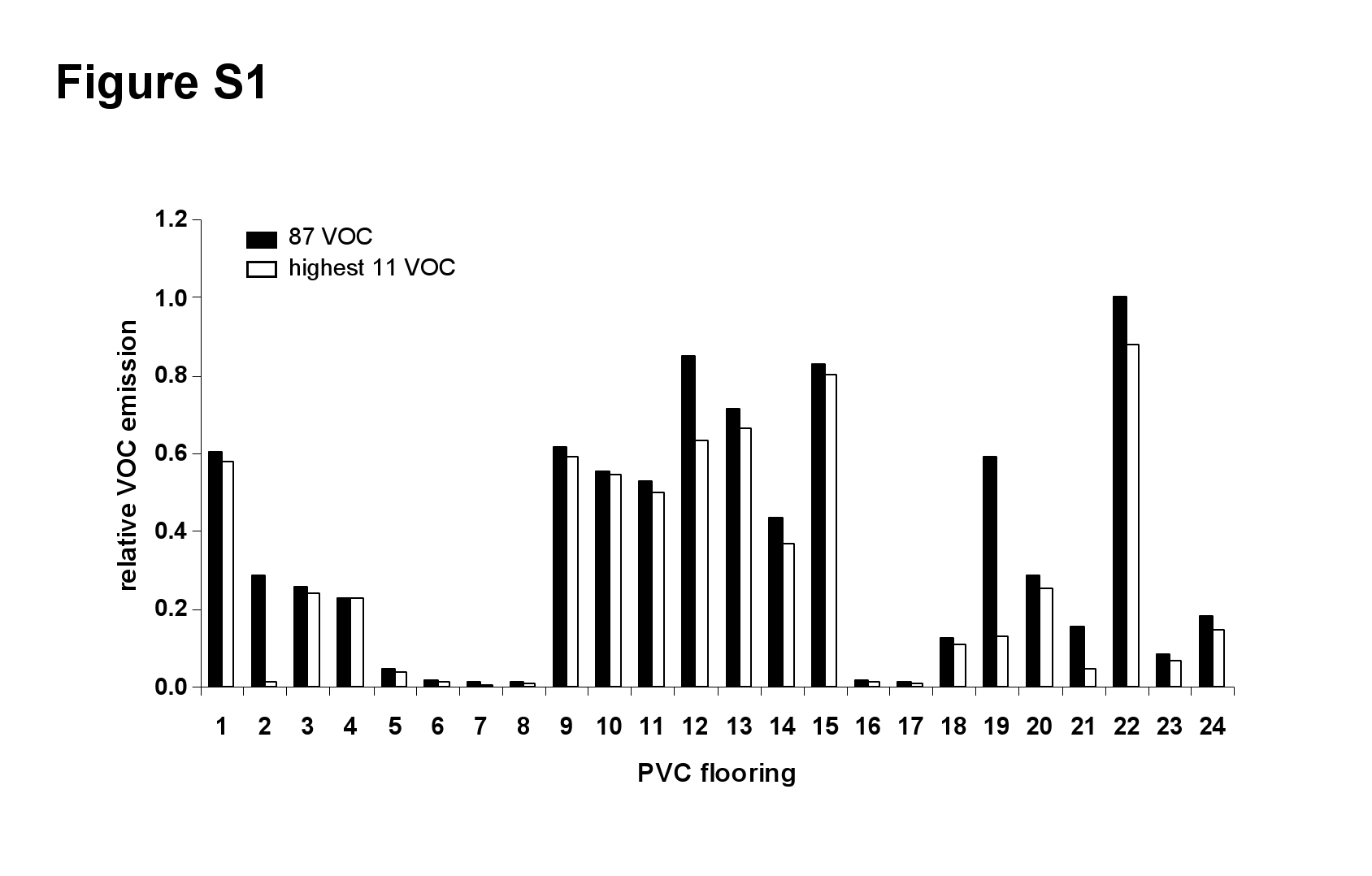

Supplement: Figure S1 — VOC emission by different PVC floorings. The measurement of VOCs by headspace sampling over 24 different PVC floorings and analysis with GC-MS revealed that the VOC emission is dominated by 11 VOCs out of 87 measured VOCs. For further in vivo experiments PVC flooring number 9 was selected. (TIF) [file pone.0039817.s001.tif]

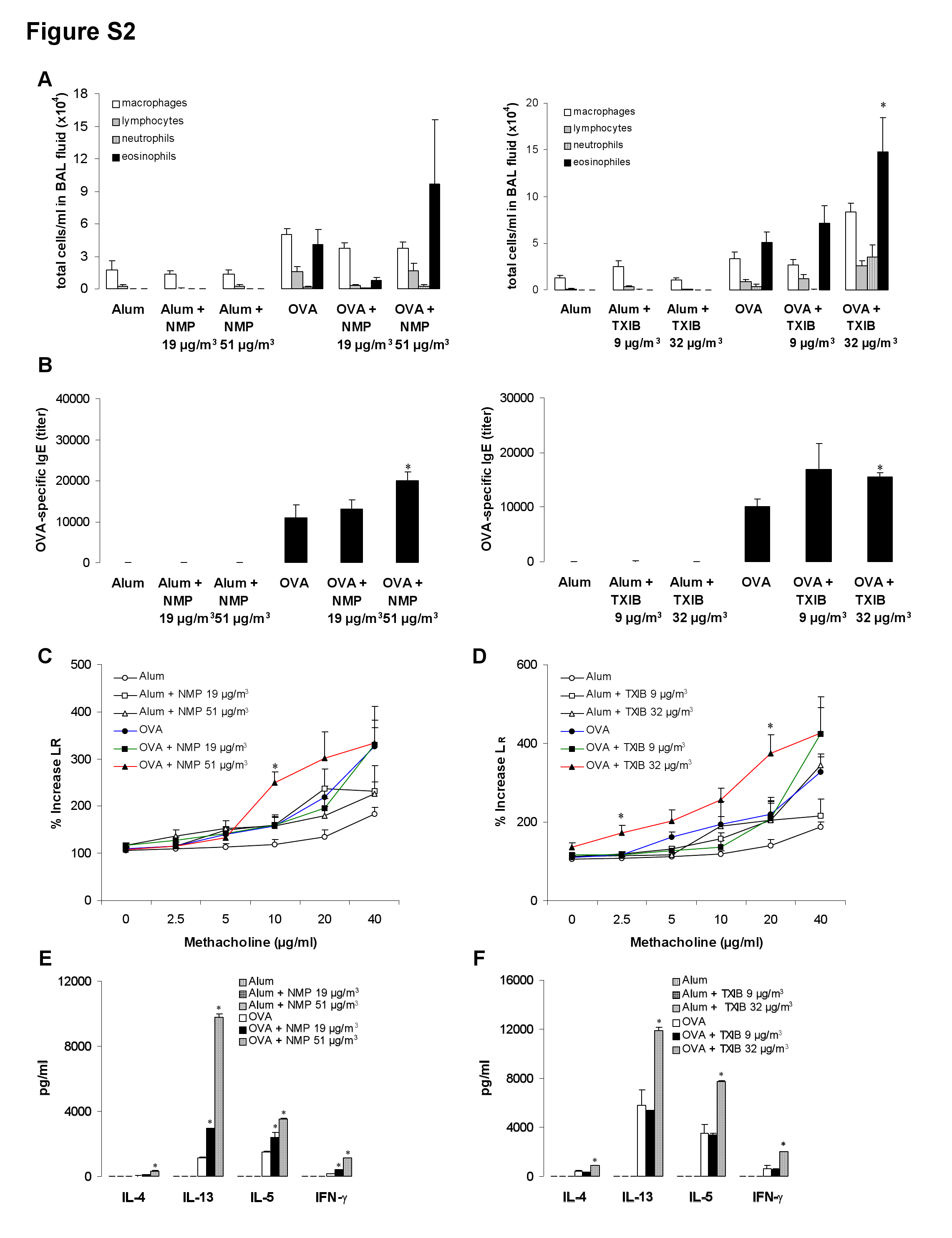

Supplement: Figure S2 — Effect of an exposure to NMP or TXIB during antigen challenge on asthma-like phenotype. To analyze the effect of NMP or TXIB exposure on an ongoing allergic inflammation Balb/c mouse were sensitized and exposed to different concentrations of the VOCs as described under Methods. Effect of exposure to NMP and TXIB on total cell numbers in BAL fluid (A), OVA-specific IgE levels (B), lung resistance (C), and cytokine production in the supernatant of OVA-re-stimulated mediastinal lymphnodes (D). Data are expressed as mean ± SEM, n ≥ 9 animals per group; *P<0.05 vs. OVA. (TIF) [file pone.0039817.s002.tif]
