# Supplementary material for: Volatile Organic Compounds Enhance Allergic Airway Inflammation in an Experimental Mouse Model
Source: PLoS One. 2012 Jul 3;7(7):e39817. doi: 10.1371/journal.pone.0039817 (PMC3389035; doi:10.1371/journal.pone.0039817)
Supplement: File S1 — Supporting Information. (DOC) [file pone.0039817.s003.doc]

**Volatile organic compounds enhance allergic airway inflammation**

**in an experimental mouse model**

Ulrike Bönisch, Alexander Böhme, Tibor Kohajda, Iljana Mögel, Nicole Schütze, Martin von Bergen, Jan C. Simon, Irina Lehmann, Tobias Polte

**SUPPORTING INFORMATION**

# METHODS

**Sampling and measurements of VOCs**

For the determination of VOCs emitted from PVC flooring, stainless steel sampling tubes filled with Tenax TA were used and preconditioned at 280 °C under a steady flow of nitrogen for 40 min. Air samples (15 l) in mice cages with installed PVC flooring on the top covers were adsorbed to these tubes. Analysis was carried out with Markes TD-100 Thermal Desorber (Markes International Ltd., USA) coupled to an Agilent 7890A gas chromatographic system with an Agilent 5975C quadrupole mass spectrometer (Agilent Technologies Inc., USA). For separation an Rxi-1ms capillary column (Restek, 60 m, 0.32 mm i.d., 1 μm df) with helium as carrier gas was used. For MS detection electron ionization (EI) with 70 eV was applied and mass fragments were detected between 33 and 360 m/z in the total ion current mode (TIC). A reference standard library (NISTMS Search 2.0, National Institute of Standards and Technology) as well as reference substances were used to identify the VOCs. For the quantification of the VOCs calibration curves were created with the use of reference substances.

Samples of PVC flooring (3 cm²) were cut into small pieces and placed in a 10 ml head space vial to determine VOC pattern. The samples were equilibrated at a defined temperature for 30 min and a 250 µl aliquot of the head space air was injected to the GC-MS.

To determine concentrations of TXIB and NMP inside the inhalation chamber air sample were collected in gas sampling bulbs (137 mL) during exposure almost daily (except on weekends). Afterwards, sampling bulbs filled with TXIB containing air were flushed with nitrogen 5.0 (= purity > 99.9999 %) at 17 mL/min for at least one hour and escaping TXIB was trapped on three succeeding sorbent tubes filled with Tenax (Chrompack, Germany). Required flow rate and time had been determined in preliminary tests employing gas sampling bulbs with known TXIB concentrations. Sampling bulbs filled with NMP were equipped with a twister (polydimethylsiloxane coated stirring bar from Gerstel, Germany), and NMP was collected from the air phase for 24 h. Subsequent analysis of the adsorbed compound was performed on a 6890N gas chromatograph equipped with a 5973N mass selective detector (both from Agilent, USA), a HP-5 MS capillary column (length: 30 m, i.d.: 0.25 mm), and a thermodesorption unit (Gerstel, Germany). Received peak areas were converted to concentrations in terms of µg/m³ employing external calibrations, which had been prepared by charging sorbent tubes with different amounts of TXIB diluted in toluene and NMP diluted in iso-propanol, respectively, followed by GC analysis as outlined above. Here, concentration ranges from 2.90 to 146 µg/m³ for both, TXIB and NMP.

**Culture and treatment of bone marrow-derived dendritic cells (BMDC)**

Bone marrow cells were collected from naïve Balb/c mice, depleted of red blood cells using ammonium chloride, and grown in RPMI medium containing 10% FCS and 20 ng/ml rmGM-CSF for 7 days. Cells were characterized by staining with an anti-CD11c– Phycoerythrin (PE) mAb and 2 x 10^6^ cells/ml were given to 24-well plates and exposed to NMP or TXIB (both 5-100 μg/m^3^) in the presence of LPS (1 μg/ml) for 18 hrs. To adjust experimental conditions of the *in vivo* mouse scenario to an *in vitro* approach employing DCs as test organisms partitioning behaviour of NMP or TXIB between air and mouse, and aqueous medium and cells had to be considered: First, accumulation of NMP/TXIB into mouse was quantified using compounds´ concentration in the air and the logarithmic octanol/air partition coefficient (log *K*_oa_). The latter forms a model parameter to describe the partitioning behaviour of NMP/TXIB between an organic phase (= mouse) and air, and was calculated using ChemProp-software . Secondly, to maintain the designated concentrations the required amount, which had to be added to the cell testing scenario was calculated considering NMP or TXIBs´ partitioning behaviour between water and an organic phase given by the logarithmic octanol/water partition coefficient (log *K*_ow_) . The supernatant was collected for cytokine measurement.

**Co-culture of OVA-pulsed DCs and CD4^+^ T cell**

Bone marrow-derived DCs (Balb/c) were pulsed for 24 h with OVA (200 µg/ml), some cells in the presence of NMP or TXIB (100 μg/m^3^, 3 h). 5 x 10^5^ OVA-pulsed DCs were co-cultured with 5 x 10^5^ separated CD4^+^ T cells (Milteny Biotek, Bergisch-Gladbach, Germany, > 95%) from DO11.10 mice which are transgenic for T cell receptor recognizing OVA specific peptide and cultured for 72 h to measure cytokine production in the supernatant .

**Transfer of OVA-pulsed DCs**

Immunization by intratracheal injection of OVA-pulsed DCs was done as described Lambrecht et al. . Briefly, bone marrow-derived DCs were pulsed for 24 h with OVA (200 µg/ml), some cells in the presence of NMP or TXIB (100 μg/m^3^). 2 x 10^6^ cells in 60 μl PBS were adoptively transferred into the trachea in anesthetized naïve Balb/c mice. From day 10 onward, mice were challenged i.n. with OVA for 3 consecutive days. AHR was measured on day 13 and mice were sacrificed the following day.

**VOC exposure of epithelial cells**

Human alveolar epithelial cells (A549, ATCC No: CCL-185; LGC Promochem, Wesel, Germany) were cultured and exposed to VOC using an air-liquid cell culture model as described previously . Briefly, tissue culture inserts (transwells) with 0.4 μm polyester membranes (Corning, NY, USA) were placed into 6- well plates and 2 x 10^5^ A549 cells were seeded into each culture insert and grown for 2 days. After replacement of culture medium with exposure medium consisting of equal volumes RPMI 1640 and CO_2_ independent medium (Invitrogen, Karlsruhe, Germany) transwells-containing dishes were transferred into pre-warmed flasks, exposed to the selected VOC (diluted in methylcyclopentane) by addition of 10 μl directly into the glass flask. For controls, 10 μl methylcyclopentane were added and cells were incubated for 24 h at 37°C.

**Western Blot**

Lung tissues from mice after short-term exposure to different concentrations of NMP (19 and 51 μg/m^3^) or TXIB (9 and 32 μg/m^3^) on two consecutive days for 6 hours were homogenized 18 hours later in 500 µl lysis buffer (2% SDS, 20% glycerol, 0.5 mM EDTA, 250 mM Tris/HCl pH 8.4 including 0.5% Complete Proteinase Inhibitor Cocktail). Human alveolar epithelial cells were washed with cold PBS and lysed by applying 200 µl lysis buffer. Protein concentrations were measured using DC Protein Assay according to the manufacturer's instructions (Bio-Rad Laboratories, Munich, Germany). Proteins were separated under denaturing conditions on 4-12% NuPAGE® Bis-Tris gradient gels (Invitrogen, Karlsruhe, Germany) and transferred onto nitrocellulose membranes (Bio-Rad Laboratories). Blotting efficiency was controlled by staining the gels with Ponceau S (Merck, Darmstadt, Germany). Membranes were blocked using ECL blocking reagent (GE Healthcare, Freiburg, Germany) and proteins were detected using GSTP-1- (Kamiya Biomedical Company) and β-actin- (Sigma-Aldrich) specific antibodies as well as horseradish peroxidase-conjugated secondary anti-mouse IgG-specific antibodies (Bio-Rad, Munich, Germany). Bands corresponding to the proteins of interest were visualised using the ECL Advance Western Blot Detection Kit (GE Healthcare) and a FluorChem® 8900 imager (Alpha Innotech, San Leandro, USA) and quantified using the free software ImageJ (NIH, USA; http://rsbweb.nih.gov/ij).

**Measurement of 8-isoprostane**

In a short-term protocol mice were exposed 6 hours/day to NMP (19 and 51 μg/m^3^) and TXIB (9 and 32 μg/m^3^) on two consecutive days. 18 hours after the last VOC exposure (independently from the protocol) lung tissues were homogenized in buffer (0.1 M phosphate, 1 mM EDTA, 10 µM indomethacin and 0.05% butylated hydroxytoluene, BHT at pH 7.4) using lysing matrix A (FastPrep®24 homogenizer, MP Biomedicals, LLC, Eschwege, Germany). Aliquots of the obtained supernatants were hydrolyzed (15% KOH) and deproteinized (ethanol containing 0.01% BHT). 8-isoprostane concentration was determined by specific immunoassay according to manufacturer’s instructions (Cayman Chemical Company, Ann Arbor, MI, USA). Protein concentration of 5 µl homogenates were measured at 700 nm using *DC* Protein Assay (Bio-Rad, München, Germany) comparing it to diluted series of bovine serum albumin as standard.

**In vivo imaging of bioluminescent signal**

Activation and localization of NF-κB in the NF-κB/luc Tg mice were visualized using an In Vivo Imaging System (IVIS Lumina, Caliper Life Sciences, Hopkinton, USA). Thirty minutes prior to imaging, the anesthetic agents (100 mg/kg ketamine and 10 mg/kg xylazine) were injected intramuscularly to obviate movement. Ten minutes later the substrate luciferin (150 mg/kg body weight) was applied intraperitoneally into Tg mice. The light emitted from the luciferase-expressing cells of the Tg mice was detected with the IVIS Lumina. To allow for quantification and normalization, the light intensity and photon counts were determined from identical areas of the lungs of live animals using Living Image software (Caliper Life Sciences, Hopkinton, USA).

**References**

1. Schüürmann G, Kühne, R., Kleint, F., Ebert, R.-U., Rothenbacher, C. and Herth, P. (1997) A software system for automatic chemical property estimation from molecular structure. Quantitative structure-activity relationships in environmental science - VII

2. Murphy KM, Heimberger AB, Loh DY (1990) Induction by antigen of intrathymic apoptosis of CD4+CD8+TCRlo thymocytes in vivo. Science 250: 1720-1723.

3. Peterson JD, Herzenberg LA, Vasquez K, Waltenbaugh C (1998) Glutathione levels in antigen-presenting cells modulate Th1 versus Th2 response patterns. Proc Natl Acad Sci U S A 95: 3071-3076.

4. Lambrecht BN, Pauwels RA, Fazekas De St Groth B (2000) Induction of rapid T cell activation, division, and recirculation by intratracheal injection of dendritic cells in a TCR transgenic model. J Immunol 164: 2937-2946.

5. Kuipers H, Soullie T, Hammad H, Willart M, Kool M, et al. (2009) Sensitization by intratracheally injected dendritic cells is independent of antigen presentation by host antigen-presenting cells. J Leukoc Biol 85: 64-70.

6. Lehmann I, Roder-Stolinski C, Nieber K, Fischader G (2008) In vitro models for the assessment of inflammatory and immuno-modulatory effects of the volatile organic compound chlorobenzene. Exp Toxicol Pathol 60: 185-193.

7. Feltens R, Mogel I, Roder-Stolinski C, Simon JC, Herberth G, et al. (2010) Chlorobenzene induces oxidative stress in human lung epithelial cells in vitro. Toxicol Appl Pharmacol 242: 100-108.
